# Individualized Internal and External Training Load Relationships in Elite Wheelchair Rugby Players

**DOI:** 10.3389/fphys.2015.00388

**Published:** 2015-12-21

**Authors:** Thomas A. W. Paulson, Barry Mason, James Rhodes, Victoria L. Goosey-Tolfrey

**Affiliations:** The Peter Harrison Centre for Disability Sport, School of Sport, Exercise and Health Sciences, Loughborough UniversityLoughborough, UK

**Keywords:** paralympic, performance, perceived exertion, exercise prescription, heart rate, speed zones

## Abstract

**Aim:** The quantification and longitudinal monitoring of athlete training load (TL) provides a scientific explanation for changes in performance and helps manage injury/illness risk. Therefore, accurate and reliable monitoring tools are essential for the optimization of athletic performance. The aim of the present study was to establish the relationship between measures of internal [heart rate (HR) and session RPE (sRPE)] and external TL specific to wheelchair rugby (WR).

**Methods:** Fourteen international WR athletes (age = 29 ± 7 years; body mass = 58.9 ± 10.9 kg) were monitored during 18 training sessions over a 3 month period during the competitive phase of the season. Activity profiles were collected during each training session using a radio-frequency based indoor tracking system (ITS). External TL was quantified by total distance (m) covered as well as time spent and distance covered in a range of classification-specific arbitrary speed zones. Banister's TRIMP, Edwards's summated HR zone (SHRZ), and Lucia's TRIMP methods were used to quantify physiological internal TL. sRPE was calculated as the product of session duration multiplied by perceived exertion using the Borg CR10 scale. Relationships between external and internal TL were examined using correlation coefficients and the 90% confidence intervals (90% CI).

**Results:** sRPE (*r* = 0.59) and all HR-based (*r* > 0.80) methods showed large and very large relationships with the total distance covered during training sessions, respectively. Large and very large correlations (*r* = 0.56 − 0.82) were also observed between all measures of internal TL and times spent and distances covered in low and moderate intensity speed zones. HR-based methods showed very large relationships with time (*r* = 0.71−0.75) and distance (*r* = 0.70−0.73) in the very high speed zone and a large relationship with the number of high intensity activities (HIA) performed (*r* = 0.56−0.62). Weaker relationships (*r* = 0.32−0.35) were observed between sRPE and all measures of high intensity activity. A large variation of individual correlation co-efficient was observed between sRPE and all external TL measures.

**Conclusion:** The current findings suggest that sRPE and HR-based internal TL measures provide a valid tool for quantifying volume of external TL during WR training but may underestimate HIA. It is recommended that both internal and external TL measures are employed for the monitoring of overall TL during court-based training in elite WR athletes.

## Introduction

Coaches and sports science practitioners continue to take an increasingly scientific approach to the prescription and monitoring of athlete training (Malone et al., 2015; McLaren et al., 2015). The longitudinal monitoring of individual training load (TL) provides a quantifiable explanation for changes in performance, ensures target doses are achieved, and helps manage illness/injury risk. External TL describes the work completed by the athlete in terms of distance, speed or power using micro-technologies including time-motion analysis, accelerometers or power-meters, respectively (Lambert and Borresen, 2010; Halson, 2014; McLaren et al., 2015). The resultant physiological or psychological stress imposed, described as internal TL, drives adaptation in the relevant metabolic, cardiovascular and neurological systems (Halson, 2014). The outcome of any training intervention is therefore the consequence of both external and internal stimuli and reliable monitoring tools are vital for the optimization of athletic performance.

Like basketball and its wheelchair-based equivalent, wheelchair rugby (WR) is a court-based, intermittent sport characterized by frequent high intensity accelerations and decelerations (Barfield et al., 2010; Rhodes et al., 2015a). Eligibility for WR classification requires a functional impairment in all four limbs and encompasses a range of physical impairments including cervical level spinal cord injury (SCI), amputees, and cerebral palsy. Recently a novel radio-frequency based indoor tracking system (ITS) has been employed to quantify the external demands of competition (Rhodes et al., 2015a) and key determinants of successful performance during WR match-play (Rhodes et al., 2015b). Athletes typically cover distances ranging between 3500–4600 m during matches (Sarro et al., 2010; Rhodes et al., 2015a) with the majority of time spent (~75%) performing low intensity activities interspersed with short, frequent bouts of high intensity activity (Rhodes et al., 2015a). The ability to reach high peak speeds and perform a greater number of high intensity activities (HIA) are key indicators of mobility associated with successful performance, as determined by team rank (Rhodes et al., 2015b).

WR squads are characterized by a large heterogeneity in athlete impairment which may result in a range of internal TL responses to the same dose of external load. Yet, training within a team sport environment is frequently prescribed on a squad-basis to develop sport-specific, technical, and tactical competences, thereby increasing the risk of non-functional over-reaching or under-training. Currently no research has investigated the use of internal TL measures during WR training in relation to commonly used measures of external TL. Barfield et al. (2010) attempted to quantify the exercise intensity of WR training sessions for a group of athletes with a cervical SCI using heart rate (HR) as a measure of internal load. However, HR is considered an ineffective tool for monitoring TL in some athletes with a cervical level SCI due the reduction in maximal HR responses (120–150 bpm^−1^) associated with impaired autonomic function (Valent et al., 2007; Paulson et al., 2013). An increasing number of non-SCI athletes now compete in WR, therefore, HR-based methods maybe suitable for these individuals. Banister's TRIMP, Edwards' summated HR zone (SHRZ), and Lucia's TRIMP are HR-based methods that have been utilized to quantify physiological load in able-bodied sports (Banister, 1991; Edwards, 1993; Lucia et al., 2003; Waldron et al., 2011; Scanlan et al., 2014). However, the use of these HR-based methods in intermittent sports may underestimate near maximal short high and very high intensity efforts due to the heavy reliance on anaerobic metabolism (Alexiou and Coutts, 2008; Akubat and Abt, 2011).

The session rating of perceived exertion (sRPE) provides an alternative method of quantifying internal TL, which describes a subjective, global rating of intensity and is the product of training duration, and perceived exertion using Borgs CR10 scale (Borg, 1998; Foster et al., 2001). Very large linear relationships are observed between HR and RPE-based methods in field and indoor intermittent sports supporting sRPE as a valid alternative for the quantification of internal TL (Impellizzeri et al., 2004; Manzi et al., 2010; Waldron et al., 2011; Scott et al., 2013; Lupo et al., 2014; Scanlan et al., 2014). Lovell et al. (2013) and Scott et al. (2013) have also observed large relationships between sRPE and external TL indices, including total distance covered, during elite Rugby League and Football training, respectively. In contrast, Weston et al. (2015) report only small relationships between overall match RPE and GPS-derived measures of external load in Australian League Football. Currently no “gold standard” method currently exists for the quantification of internal TL during high intensity/intermittent activities representative of WR. The aim of this study was to establish the relationship between traditional measures of internal TL (HR and sRPE) and external TL measures specific to WR.

## Methods

### Participants

Fourteen international WR players (age = 29 ± 7 years; body mass = 58.9 ± 10.9 kg; time in sport = 9 ± 2 years; training hours = 9 ± 2 h.wk^−1^; *n* = 1 female) with a cervical SCI (*n* = 9) and non-SCI (*n* = 5) volunteered to participate in the current study. Ethical approval for the study was obtained through Loughborough University's ethics committee. Prior to participation, all players provided their written, informed consent.

### Design

The study employed a single cohort observation with data collected during a total of 18 WR training sessions performed over a 3 month period during the competitive phase of the season. Prior to the training phase all participants performed an initial laboratory exercise test for the determination of resting (HR_rest_) and peak (HR_peak_) HR and peak oxygen uptake ($\dot{V}$O_2peak_). During training sessions external and internal TL data were collected for all athletes using the ITS and sRPE, respectively. HR was only collected during training from the non-SCI players. All training sessions were performed at the same indoor venue on wooden sprung flooring. Data were only analyzed for individuals completing whole training sessions.

### Submaximal test and graded-exercise test to exhaustion (GXT)

HR_rest_ was determined following a 10-min rest in a semi-supine position using radio telemetry (Polar PE 4000, Kempele, Finland). All participants' performed the tests in their competition sports wheelchair on a motorized treadmill (HP Cosmos, Traunstein, Germany). The submaximal test and GXT were performed according to the protocols described by Leicht et al. (2012). Briefly, participants performed six to eight submaximal constant-load 4-min exercise blocks at ascending speeds at a fixed gradient of 1.0%, in order to elicit physiological responses covering a range from 40 to 80% $\dot{V}$O_2peak_ (Leicht et al., 2012). This was followed by a 15-min passive recovery. The gradient at the start of the GXT was 1.0% with subsequent increases of 0.1% every 40 s to ensure a minimum GXT duration of ~8 min. After the GXT, participants recovered actively at a low intensity (1.2 ms^−1^) at a 1.0% gradient for 5 min. Participants then performed a verification test, designed as a test to exhaustion at the same constant speed but 0.1% higher than the maximal gradient achieved during the GXT. The GXT and the verification test were terminated when participants were unable to maintain the speed of the treadmill. HR was measured throughout the test with the highest 5 s rolling average used to establish HR_peak_. On-line respiratory gas analysis was carried out throughout the GXT and verification stage via a breath-by-breath system (Cortex metalyser 3B, Cortex, Leipzig, Germany). Before the test, gases were calibrated according to the manufacturer's recommendations using a 2-point calibration (O_2_ = 17.0%, CO_2_ = 5.0% against room air) and volumes with a 3-L syringe at flow rates of 0.5–3.0 L·s^−1^. Breath-by-breath data allowed the highest 30 s rolling average $\dot{V}$O_2_ value recorded and was taken as the $\dot{V}$O_2peak_.

### External TL

Activity profiles were quantified during each training session using a radio-frequency based ITS (Ubisense, Cambridge, UK) described previously (Rhodes et al., 2014; Perrat et al., 2015). Each participant was equipped with a small lightweight tag (25 g), which was attached on or near the foot-strap of athletes own rugby wheelchairs. Tags communicate wirelessly at a frequency of 8 Hz via ultra wideband radio signals with six sensors elevated around the perimeter of the court (28 × 15 m) to provide time and location data in three dimensions. The reliability of tags operating at this sampling frequency range between a coefficient of variation of 0.5% for distance covered and mean speed reached and never exceeded 2.0% for peak speed detection (Rhodes et al., 2014).

External TL was quantified by the total distance (m) covered during each training session. The time spent and distance covered in a range of classification-specific arbitrary speed zones, determined by the mean, peak speed (V_max_) of each class, as previously defined by Rhodes et al. (2015a) were also reported. These speed zones were based on a percentage of the peak speed (%V_max_) for each classification group and were categorized as the following intensities: zone 1 = very low speed (≤ 20%V_max_), zone 2 = low speed (21–50%V_max_), zone 3 = moderate speed (51–80%V_max_), zone 4 = high speed (81–95%V_max_), and zone 5 = very high speed (>95%V_max_). The number of HIA, as defined by the frequency of bouts performed in both high and very high speed zones, were also recorded.

### Internal TL

#### HR-based methods

During training HR was collected via a Polar team system (Polar Team^2^, Kempele, Finland) sampling at 5 s intervals. This HR data were incorporated into the Banister's TRIMP (Banister, 1991), Edwards SHRZ (Edwards, 1993) and Lucia's TRIMP to provide physiological measures of internal TL and are quantified in arbitrary units (AU). Banisters' TRIMP combines predetermined, individualized HR_peak_ and HR_rest_ measures, as well as the average HR during training (HR_ex_). The activity intensity is weighted using a fixed exponential relationship between changes in HR and blood lactate concentration during incremental exercise (Banister, 1991). The formula to determine TL in males using the TRIMP model proposed by Banister is as follows:

$$\begin{array}{l} \text{TRIMP training load }(\text{AU})=[\text{duration }(\text{min})]({\text{HR}}_{\text{ex}\;-}{\text{HR}}_{\text{rest}})/\\ \;({\text{HR}}_{\text{peak}}-{\text{HR}}_{\text{rest}})\times 0.64e^{\text{1}.\text{92x}}\\ \text{where }e=2.712,\;\text{and x}=({\text{HR}}_{\text{ex}}-{\text{HR}}_{\text{rest}})/\\ \;({\text{HR}}_{\text{peak}}-{\text{HR}}_{\text{rest}}). \end{array}$$

The SHRZ model proposed by Edwards determines internal TL by multiplying the accumulated training duration in five discrete HR zones relative to HR_peak_ by a coefficient relative to each zone and summating the results. The formula to determine TL using the SHRZ model is represented as:

$$\begin{array}{l} \text{SHRZ training load }(\text{AU})=(\text{duration in zone l }\times \text{ l})\\ \;+(\text{duration in zone 2 }\times \text{ 2})\\ \;+(\text{duration in zone 3 }\times \text{ 3})\\ \;+(\text{duration in zone 4 }\times \text{ 4})\\ \;+(\text{duration in zone 5 }\times \text{ 5})\\ \text{where zone }1=50-60\%\;{\text{HR}}_{\text{peak}};\\ \text{ zone }2=60-70\%\;{\text{HR}}_{\text{peak}};\\ \text{ zone }3=70-80\%\;{\text{HR}}_{\text{peak}};\\ \text{ zone }4=80-90\%\;{\text{HR}}_{\text{peak}};\\ \text{and zone }5=90-100\%\;{\text{HR}}_{\text{peak}}. \end{array}$$

Lucia's TRIMP method was calculated by multiplying the time spent in three different HR zones (zone 1 = below the ventilatory threshold, zone 2 = between the ventilatory threshold and the compensation point, zone 3 = above the respiratory compensation point) by a co-efficient for each zone (zone 1 = 1, zone 2 = 2, zone 3 = 3) and summating the results. HR zones are therefore defined on individual parameters obtained in the laboratory (Lucia et al., 2003). Lactate thresholds were employed as previously indicated (Impellizzeri et al., 2004) due to the more frequent threshold determination using BLa over ventilatory data in wheelchair athletes reported by Leicht et al. (2014).

### Session RPE

The session RPE represents a single global rating of the intensity of a training session as described previously by Foster et al. (2001). Prior to the study all training participants were familiarized with the Borg CR10 scales and the associated verbal anchors (Borg, 1998). Within 30 min of a training session being completed participants were shown the scale and asked to provide a rating of the overall perceived intensity of the session. The sRPE was then calculated by multiplying the duration of the session in minutes by the individual RPE for that training session and was again presented as AU.

### Statistical analyses

Participants completing < 5 training sessions were excluded from the statistical analysis leaving a total number of 78 observations from nine participants (*n* = 6 cervical SCI). All data were analyzed using the Statistical package for the Social Sciences (SPSS version 21.0, Chicago, Illinois, USA). The mean ± SD were calculated for each measure of external and internal TL. A within-measures design was used to determine if high internal load measures (Banister's, Edwards, Lucia's, sRPE) were associated with higher ITS-derived TL measures for the whole group as described previously (Bland and Altman, 1995). Confidence intervals (90% CI) for the within-player correlations were calculated. Individual relationships between external and internal TL measures were examined using Pearson correlation coefficients and the 90% CI. The magnitude of all correlations were categorized as trivial (*r* < 0.1), small (*r* = 0.1–0.3), moderate (*r* = 0.3–0.5), large (0.5–0.7), very large (*r* = 0.7–0.9), nearly perfect (*r* > 0.9), and perfect (*r* = 1; Hopkins et al., 2009). Statistical significance was set at *P* < 0.05.

## Results

The mean duration of all training sessions was 143 ± 40 min and ranged from 84 to 230 min. The mean external load measures of all training sessions are presented in Table 1. Mean internal TL was 97 ± 38 AU (Banisters), 310 ± 119 AU (Edwards), 247 ± 74 (Lucia's), and 934 ± 359 AU (sRPE). A large correlation was found between sRPE and both Banisters TRIMP (*r* = 0.62), Edwards SHRZ (*r* = 0.64). In addition, a very large correlation was found between sRPE and Lucia's TRIMP (*r* = 0.81).

**Table 1:** **Descriptive statistics of external load measures during wheelchair rugby training sessions measured by the ITS (*n* = 9)**.

	**Mean (SD)**	**Range**	**% of training session**
Total distance (m)	4511 (1666)	1678–8694	–
Time in Zone 1	24:23 (12:13)	05:27–52:51	38.4
Time in Zone 2	24:05 (11:01)	07:41–54:21	38.0
Time in Zone 3	11:49 (04:08)	04:02–25:12	18.6
Time in Zone 4	02:23 (01:22)	00:34–06:00	3.8
Time in Zone 5	00:38 (00:33)	00:00–02:46	1.0
Distance in Zone 1	458 (193)	113–962	10.2
Distance in Zone 2	1781 (851)	589–4113	39.5
Distance in Zone 3	1655 (597)	569–3463	36.7
Distance in Zone 4	462 (269)	107–1164	10.2
Distance in Zone 5	147 (134)	0–674	3.3
HIA (*n*)	53 (29)	14–127	–

All distances (m) and times (mm:ss).

HR and sRPE-based methods of internal TL showed a very large and large correlation with the total distance covered during training sessions, respectively. Table 2 demonstrates the relationship between measures of external TL associated with exercise intensity and internal TL. Very large correlations were observed between Banisters TRIMP, Edwards SHRZ and Lucia's TRIMP and the times spent and distances covered in speed zones 2, 3, and 5. Large, significant correlations were observed between sRPE and the time spent and distance covered in zones 2 and 3. All HR-based methods demonstrated a large relationship (0.56–0.62) with the number of HIA performed. No significant correlation was identified between the number of HIA performed and sRPE.

**Table 2:** **Within-individual correlation coefficients (90% confidence interval) for relationship between intensity measures of external load and internal training load**.

**External load**	**Internal load**			
	**Banisters TRIMP (*n* = 31)**	**Edwards SHRZ (*n* = 31)**	**Lucias TRIMP (*n* = 31)**	**sRPE (*n* = 78)**
Total Distance	0.81^**^ (0.67–0.89)	0.84^**^ (0.72–0.91)	0.82^**^ (0.69–0.90)	0.59^*^ (0.47–0.70)
Time in Zone 1	0.37 (0.08–0.60)	0.40 (0.22–0.63)	0.39 (0.10–0.62)	0.37 (0.20–0.53)
Time in Zone 2	0.85^**^ (0.74–0.92)	0.87^**^ (0.77–0.93)	0.83^**^ (0.69–0.90)	0.56^*^ (0.42–0.68)
Time in Zone 3	0.66^*^ (0.46–0.81)	0.72^**^ (0.53–0.84)	0.75^**^ (0.59–0.86)	0.59^*^ (0.45–0.70)
Time in Zone 4	0.41 (0.12–0.63)	0.41 (0.13–0.63)	0.37 (0.08–0.60)	0.22 (0.03–0.39)
Time in Zone 5	0.75^**^ (0.58–0.86)	0.75^**^ (0.57–0.85)	0.71^**^ (0.52–0.83)	0.33 (0.15–0.49)
Distance in Zone 1	0.52^*^ (0.26–0.71)	0.52^*^ (0.26–0.71)	0.51^*^ (0.25–0.71)	0.45 (0.28–0.59)
Distance in Zone 2	0.82^**^ (0.69–0.90)	0.84^**^ (0.72–0.91)	0.81^**^ (0.67–0.89)	0.56^*^ (0.42–0.68)
Distance in Zone 3	0.67^*^ (0.46–0.81)	0.72^**^ (0.53–0.84)	0.74^**^ (0.56–0.85)	0.58^*^ (0.55–0.69)
Distance in Zone 4	0.43 (0.15–0.65)	0.43 (0.15–0.65)	0.39 (0.10–0.62)	0.22 (0.03–0.39)
Distance in Zone 5	0.72^**^ (0.53–0.84)	0.73^**^ (0.54–0.84)	0.70^*^ (0.10–0.62)	0.35 (0.18–0.51)
No. of HIA	0.62^*^ (0.39–0.78)	0.61^*^ (0.38–0.77)	0.56^*^ (0.50–0.83)	0.32 (0.14–0.48)

* Large within-individual correlation (r = 0.5–0.7).

** Very large within-individual correlation (r = 0.7–0.9).

Individual correlation coefficients between sRPE and measures of external TL are presented in Table 3. The only measures of external TL that demonstrated a positive correlation with sRPE for all individuals were the times spent in speed zone 2 and the distances covered in speed zones 1 and 2.

**Table 3:** **Individual correlation coefficients between sRPE and measures of external training load**.

**Participant**			**Time in speed zones**					**Distance in speed zones**					
	***n***	***TD***	**1**	**2**	**3**	**4**	**5**	**1**	**2**	**3**	**4**	**5**	**HIA**
1	8	0.44	0.81	0.65	−0.22	−0.43	0.32	0.80	0.63	−0.25	−0.42	0.29	−0.54
2	11	0.52	0.50	0.46	0.54	0.04	0.44	0.59	0.41	0.55	0.04	0.53	0.07
3	7	−0.03	0.62	0.43	−0.03	−0.69	0.12	0.42	0.41	−0.28	−0.69	0.02	−0.70
4	8	0.82	0.26	0.79	0.77	0.76	0.82	0.27	0.79	0.79	0.77	0.78	0.72
5	7	0.39	0.49	0.62	0.46	−0.58	−0.13	0.56	0.58	0.40	−0.58	0.06	−0.55
6	7	0.61	0.83	0.71	0.47	−0.39	0.60	0.81	0.72	0.43	−0.40	0.62	−0.09
7	16	0.38	0.23	0.24	0.56	0.04	−0.08	0.19	0.25	0.57	0.04	−0.05	0.17
8	9	0.70	0.71	0.74	0.55	0.28	0.33	0.72	0.73	0.43	0.25	0.33	0.16
9	5	0.82	0.97	0.67	0.68	0.85	0.67	0.96	0.67	0.69	0.83	0.67	0.68
Min	5	−0.03	0.23	0.24	−0.22	−0.69	−0.13	0.19	0.25	−0.28	−0.69	−0.05	−0.70
Max	16	0.82	0.97	0.79	0.77	0.85	0.82	0.96	0.79	0.79	0.83	0.78	0.72
Range	11	0.85	0.74	0.55	0.99	1.54	0.95	0.77	0.54	1.07	1.52	0.83	1.42

## Discussion

The individualization of athlete training is vital to optimize physical preparation within a team environment. Reliable and valid tools are required to accurately quantify intermittent, court-based TL involving athletes with the range of physical impairments displayed in WR. An interesting finding of the current study was the large relationships between all internal TL measures and total distance covered during training as previously observed in the able-bodied sports of elite football (Casamichana et al., 2013; Scott et al., 2013) and rugby league (Lovell et al., 2013). All internal TL measures demonstrated large or very large correlations with time spent and distance covered in speed zones 2 (low) and 3 (moderate). Also in accordance with previous findings (Casamichana et al., 2013; Scott et al., 2013), weaker relationships were observed between internal TL and external TL measures of high intensity training, including the number of HIA performed. The current observations suggest sRPE and HR-based measures of internal TL provide a valid tool for quantifying volume measures of external TL during WR training but sRPE may underestimate high intensity training doses. Large ranges in within-individual sRPE-external TL relationships suggest a variety of perceptual cues are responsible for determining sRPE during WR training. It is recommended that both internal and external TL measures are employed for the monitoring of overall TL during court-based training in elite WR athletes.

Coaches and Sport Science practitioners prescribe external TLs to replicate or exceed competition intensities and induce physiological and/or psychological stress (i.e. internal TL) that drives subsequent training adaptation. The use of HR in intermittent sports is less straightforward than for endurance/aerobic-based sports, due to the heavy reliance on anaerobic metabolism and the associated delay in HR response with short duration, high intensity efforts (Alexiou and Coutts, 2008; Akubat and Abt, 2011). sRPE has been proposed as a cost-effective alternative to HR-based methods as a global measure of training intensity that may more accurately quantify internal TL in intermittent sports. In accordance with previous findings in football (Impellizzeri et al., 2004; Alexiou and Coutts, 2008), rugby union (Waldron et al., 2011), and basketball (Manzi et al., 2010), Table 2 displays large and very large relationships between sRPE and Banisters TRIMP, Edward's SHRZ, and Lucia's TRIMP.

An interesting finding of the present study was the large relationships between all internal TL measures and total distance covered during intermittent, court-based WR training. Scott et al. (2013) previously observed very large (*r* = 0.71–0.84) correlations between internal TL measures (sRPE, Bansiters TRIMP, Edwards SHRZ) and total distance covered and volume of low speed activity during in-season training of 15 professional football players. Similarly, Casamichana et al. (2013) found large to very large associations between total distance and both sRPE and Edwards SHRZ in 28 semi-professional football players over 44 training sessions. Lovell et al. (2013) investigated the validity of sRPE for quantifying overall TL in 32 professional rugby league players. A very large correlation was observed between sRPE and total distance (*r* = 0.69–0.80) in conditioning, skills-conditioning, and speed-based training (Lovell et al., 2013). A large significant correlation was also observed in the present study between the time spent and distance covered in low and moderate speed zones, with stronger relationships between HR-based methods (*r* = 0.63–0.84) than sRPE (*r* = 0.54–0.59). The present findings support both internal TL variants as a marker of volume (total distance covered) and low/moderate intensity activity. This is significant as WR match-play and training are frequently characterized by large volumes of low intensity movements (~75%) interspersed with short, frequent bouts of high intensity activity (Sarro et al., 2010; Rhodes et al., 2015a).

Weaker relationships were observed between sRPE (~0.30) distance covered and time spent in high (zone 4) and very high (zone 5) speed zones vs. all HR-based methods. Previously, sRPE has been found to display weaker relationships to high/very high speed running activity (*r* = 0.40–0.67) in professional football (Scott et al., 2013) and high intensity-based measures of rugby league TL (Lovell et al., 2013). As the criterion speed of external TL increases, the strength of relationship to sRPE becomes weaker (Scott et al., 2013). This may represent the small window in which RPE can change (1–10) and the lack of sensitivity to small manipulations in training intensity. Also high speed activities interspersed with long periods of rest may reduce RPE despite high activity levels. Typically less than 5% of time during WR match-play is spent at speeds above 80% Vmax (Rhodes et al., 2015a,b). However, the sRPE-based relationships described above may under-estimate large volumes of time spent/distance covered in high or very high speed zones that accumulate during intensive training periods.

A novel finding of the present work was the large variation observed in individual relationships between sRPE and all external TL measures times spent in low intensity speed zones (zone 2) and the distances covered in very low (zone 1) and low intensity speed zones (zone 2). Perceived exertion is a subjective global rating of intensity governed by a multitude of physiological, psychological, and environmental perceptual cues (Hampson et al., 2001). While the subjective range of intensity (from min to max effort) is known to be equal between individuals, the dominant cues determining perceptions of effort may differ greatly (Lambert and Borresen, 2010). Interestingly, Weston et al. (2015) observed only small to moderate relationships between differentiated and overall sRPE and match-play movement demands in Australian League Football. By analysing the intra-individual correlation co-efficient it is clear a wide range of relationships are present between sRPE and external load measures (i.e. total distance *r* = −0.03–0.82). All participants were familiarized in using the scale prior to the study using standardized instructions (Borg, 1998). However, factors including technical role on court, accumulated fatigue, or psychological stress could all influence an individual athlete's perception of effort during a training session. As previously described, players performing very defensive roles on court may spend a large portion of training performing low-volume activity, including blocking maneuvers, with a high physiological cost. Therefore, baselines of RPE for distinct training intensities should be established by practitioners prior to any longitudinal monitoring in order to gain an insight into intra-individual variations in RPE.

A limitation of the current methodology was that no distinction was made between on-court training modes during the correlation analysis. Weaving et al. (2014) recently employed principle component analysis to explore the influence of training modality on relationships between TL measures during sport-specific training modes 32 rugby league players. For skills training, external measures of body load and total impacts explained the greatest proportion of variance in TL (Weaving et al., 2014). Internal measures of sRPE and Banisters TRIMP explained the greatest variance in speed-based training (Weaving et al., 2014). HIA including jumping, turning, physical contact, or resistance training may be recorded as low speed activity but demand a high physiological load (Scott et al., 2013; Weaving et al., 2014). The metabolic cost of sport-specific skills, including dribbling and kicking in football and tackling in rugby, is also greater than running alone at the same speed (Scott et al., 2013). It is therefore recommended that external TL data are considered within the context of the training environment and a combination of internal and external load measures employed to accurately quantify across training modes (Weaving et al., 2014). Future research should explore the individual internal TL responses to external TL doses experienced during individual WR-specific training drills with a larger cohort of participants.

In conclusion, methods for quantifying external TL, particularly the no. of HIA performed, should always be employed for monitoring overall TL in elite WR athletes. sRPE provides a valid alternative to HR-based methods for assessing distance covered and low to moderate intensity activity in individuals with an impaired HR response. However, sRPE-based measures may underestimate the dose of external TL performed at high or very high intensities. The intra-individual relationships between external TL measures and sRPE should be assessed for each athlete prior to performing any systematic longitudinal monitoring.

### Conflict of interest statement

The authors declare that the research was conducted in the absence of any commercial or financial relationships that could be construed as a potential conflict of interest.
